# Progression of the smoking epidemic in high-income regions and its effects on male-female survival differences: a cohort-by-age analysis of 17 countries

**DOI:** 10.1186/s12889-020-8148-4

**Published:** 2020-01-10

**Authors:** Maarten Wensink, Jesús-Adrián Alvarez, Silvia Rizzi, Fanny Janssen, Rune Lindahl-Jacobsen

**Affiliations:** 1 Interdisciplinary Centre on Population Dynamics, University of Southern Denmark, Odense, Denmark; 2 Department of Public Health, University of Southern Denmark, Odense, Denmark; 3 Population Research Centre, Faculty of Spatial Sciences, University of Groningen, Groningen, The Netherlands; 4 Netherlands Interdisciplinary Demographic Institute, The Hague, The Netherlands

**Keywords:** Sex differences, Life expectancy, Smoking epidemic, Mortality, Health inequality

## Abstract

**Background:**

Of all lifestyle behaviours, smoking caused the most deaths in the last century. Because of the time lag between the act of smoking and dying from smoking, and because males generally take up smoking before females do, male and female smoking epidemiology often follows a typical double wave pattern dubbed the ‘smoking epidemic’. How are male and female deaths from this epidemic differentially progressing in high-income regions on a cohort-by-age basis? How have they affected male-female survival differences?

**Methods:**

We used data for the period 1950–2015 from the WHO Mortality Database and the Human Mortality Database on three geographic regions that have progressed most into the smoking epidemic: high-income North America, high-income Europe and high-income Oceania. We examined changes in smoking-attributable mortality fractions as estimated by the Preston-Glei-Wilmoth method by age (ages 50–85) across birth cohorts 1870–1965. We used these to trace sex differences with and without smoking-attributable mortality in period life expectancy between ages 50 and 85.

**Results:**

In all three high-income regions, smoking explained up to 50% of sex differences in period life expectancy between ages 50 and 85 over the study period. These sex differences have declined since at least 1980, driven by smoking-attributable mortality, which tended to decline in males and increase in females overall. Thus, there was a convergence between sexes across recent cohorts. While smoking-attributable mortality was still increasing for older female cohorts, it was declining for females in the more recent cohorts in the US and Europe, as well as for males in all three regions.

**Conclusions:**

The smoking epidemic contributed substantially to the male-female survival gap and to the recent narrowing of that gap in high-income North America, high-income Europe and high-income Oceania. The precipitous decline in smoking-attributable mortality in recent cohorts bodes somewhat hopeful. Yet, smoking-attributable mortality remains high, and therefore cause for concern.

## Background

According to the Global Burden of Disease Study, in 2015 worldwide, one out of ten deaths was due to smoking [1]. The same data suggest that smoking is the single most important killer in the world with nearly twice as many victims as the 5% deaths from AIDS, malaria and tuberculosis combined [2]. As such, the smoking epidemic is having a huge impact on the world population and on the individual risk of transitioning to the worst possible health condition, i.e. to die [3].

**Fig. 1:**
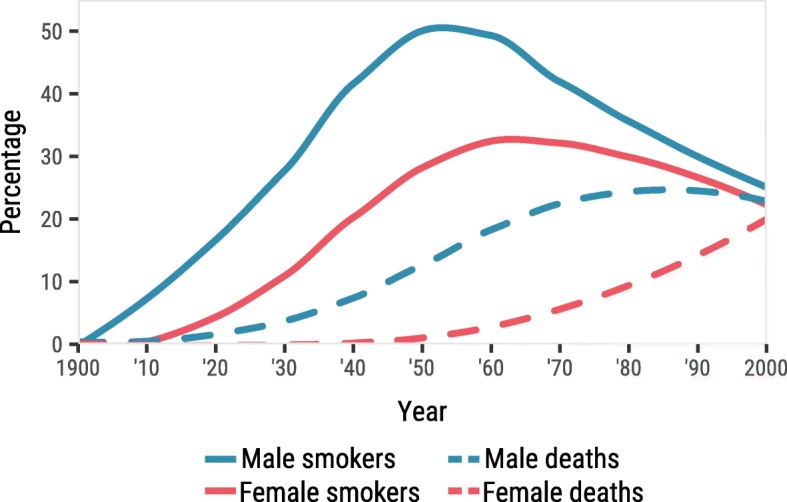
Schematic diagram of smoking epidemic, after reference 5. Males (in blue) take up smoking (solid line) at a steady pace until smoking-attributable mortality surges (dashed line) and the proportion smoking starts to decline. Females (in red) take up smoking later than males and reach a lower maximum proportion smoking. Smoking-attributable mortality in females is the last to increase to significant proportions. An essential feature of the model is the large time gap between the act of smoking and dying from it

The enormous increase and subsequent decline in smoking prevalence and later smoking-attributable mortality, and sex differences therein, has been described in detail and termed the ‘smoking epidemic’ [4, 5]. The smoking epidemic model describes that men in high-income countries (particularly the Anglo-Saxon countries) were the first to take up smoking and that smoking-attributable mortality rose some three decades after the rise in smoking prevalence. Women began to smoke later in time than males. Attention for the negative health effects of smoking and associated prevention campaigns led the proportion of males that smoke to decline and the peak in the smoking prevalence among women to be considerably lower than for men. Because of the various time lags, there is a stage where the proportion of males dying from smoking begins to decline, but the proportion of females continues to rise (Fig. 1) [4, 5].

In the 1950s, over 50% of males in the United States (US) were smokers. By 2015, this had changed to less than 20%. For females, these numbers were 24% in the 1950s, versus 12–15% currently [6–11]. For Europe this pattern was similar [7, 8]. Because the percentages of smokers for males versus females are first divergent, then convergent, in line with the theoretical model outlined above and in Fig. 1, and because high-income countries have progressed furthest into the smoking epidemic, we aimed to chart the progression of deaths from smoking in high-income North-America, high-income Europe and high-income Oceania, as well as the way these deaths have influenced male-female survival differences.

The cohort perspective has been helpful in understanding the unfolding of the smoking epidemic [12]. Also, smoking has been shown to be a significant driver of sex differences in survival [13–18]. Hence, we performed a cohort-by-age analysis of smoking-attributable mortality and investigated its effect on male-female life expectancy differences. We hypothesized that the smoking epidemic may have been the main contributor to the widening and subsequent narrowing of the male-female survival gap in high-income regions in the second half of the twentieth century. We also hypothesized that the cohort perspective could reveal important aspects of the smoking epidemic.

## Methods

All-cause death rates and person-years at risk by age, sex, year and country were retrieved from the Human Mortality Database (HMD) [19], which collects these data from national registries worldwide and, after quality control, publishes these in a uniform format [20]. To estimate smoking-attributable mortality (see below), we used lung cancer deaths, defined as malignant neoplasms of trachea, bronchus and lung classified according to the International Statistical Classification of Diseases, Injuries and Causes of Death versions 7 through 10 (ICD-7: 162, 163; ICD-8 and ICD-9: 162; ICD-10: C33, C34). Death counts from lung cancer were retrieved from the World Health Organization (WHO) Mortality Database [21]. Their cause-of-death statistics come from national vital registration systems. The information is compiled by the national authority and submitted to WHO every year. WHO verifies that the data submitted are coded with the official ICD codes [21].

Mortality data by age (50–85 years) and sex for the populations of 17 high-income countries during the period 1950–2015 were used. The focus on age 50 and over stems from the technique that we used to indirectly estimate smoking-attributable mortality (see below). This method relies on deaths from lung cancer, which are generally rare below age 50 [22]. Furthermore, previous research has shown that most of male-female mortality differences are concentrated between ages 50 and 70 [13].

The method that we used to estimate smoking-attributable mortality, the Preston-Glei-Wilmoth (PGW) method [23] (see below), was based on 20 high-income countries from around the world. We used much the same data set, but we added recent data and excluded three countries. We excluded Iceland because of its geographical location between Europe and North America. We excluded countries that spent much of the 1950–2015 period behind the Iron Curtain, because the countries missed out on the cardiovascular revolution for decades, a phenomenon  sometimes called the state socialist syndrome [24]. This led us to exclude Hungary. Japan was excluded because of its atypical smoking history [25]. For Portugal, cause-specific mortality data were not available for the period 2004–2006. We imputed death rates for males and females for Portugal during the period 2004–2006 (3 observations per sex in total, details in Additional file 1) by taking into account all the remaining information about death rates since 1950. We use a non-parametric method based on random forest algorithms to perform the imputation (“missForest” R package, version 1.4 [26])”. The 17 remaining countries were grouped into three geographical categories: high-income North America (US and Canada), high-income Europe (Austria, Belgium, Denmark, Finland, France, Italy, the Netherlands, Norway, Portugal, Spain, Sweden, Switzerland and the UK), and high-income Oceania (Australia and New Zealand).

The Preston-Glei-Wilmoth (PGW) method [23] was used to appraise the proportion of deaths attributable to smoking. This method assumes that “after adjusting for sex and age, smoking is the only source of variation in lung cancer death rates in the populations under consideration” [23]. It estimates smoking attributable deaths indirectly by assuming that lung cancer rates of smokers and never-smokers match those observed among individuals in the Cancer Prevention Study II in the US. The model then uses negative binomial regression to model smoking attributable mortality from causes other than lung cancer as a function of lung cancer mortality [23]. An analysis of deviance to assess the model fit is included in the Additional file 1.

We then smoothed smoking-attributable mortality estimates with the penalized composite link model [27], giving year-by-age estimates of the proportions of death due to smoking for all 17 countries for males and females over age 50. We used such year-by-age estimates to construct birth-cohorts’ mortality profiles between 1870 and 1965 for high-income North America, high-income Europe and high-income Oceania [28]. Thus we obtained the proportion of smoking-attributable mortality by sex, cohort and age (cohorts 1870–1965).

To shed light on how smoking-attributable mortality affected period life expectancy, the way in which the sex gap in survival is often analyzed [13], we calculated period life expectancy between ages 50 and 85 (*e*_50 ∣ 85_, the average number of years lived between ages 50 and 85) over the period 1950–2015 following standard demographic procedures [29]. This measure is defined as follows:

$$ {e}_{50\mid 85}=\frac{\sum \limits_{50}^{85}l(x)}{l(50)}, $$

where *l*(*x*) denotes number of survivors at age x. We applied the life table calculations to all-cause mortality rates (including smoking-attributable mortality) and to non-smoking-attributable mortality rates (excluding smoking-attributable mortality) for the three regions and the different years. From those results we calculated male-female *e*_50 ∣ 85_ differences for all regions and periods.

All analyses were run on R version 3.6.

## Results

Between 1950 and 2015, the smoking epidemic caused a total of 39 million deaths at ages 50–85 in the three high-income geographical locations. Of these, 29 million deaths were men and 10 million were women. The largest numbers of deaths attributable to smoking were in high-income North America with 13 million men and 7 million women, followed by high-income Europe with 15 million and 3 million, respectively. In high-income Oceania these numbers were 0.7 million and 0.2 million.

**Fig. 2:**
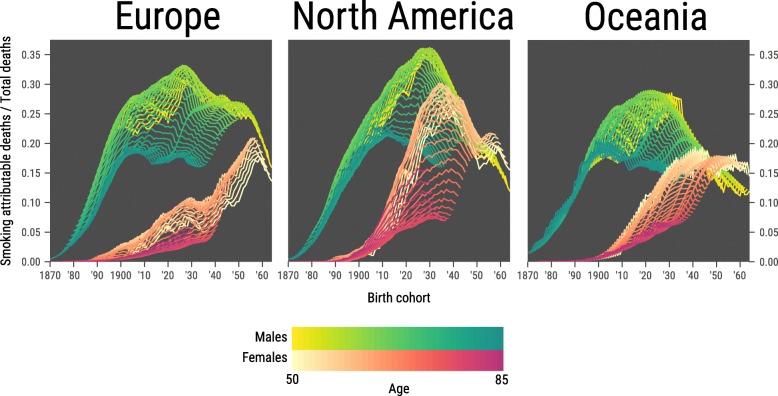
Cohort-by-age analysis of the proportion of overall mortality that is attributed to smoking. Each birth cohort is on a single vertical line. For males, ages are shaded from yellow (age 50) to turquoise (age 85). For females, ages are shaded from beige (age 50) to fuchsia (age 85). The more recent a cohort, the smaller the number of age groups for which data are available (recent cohorts have not yet reached the higher ages). Results given for high-income Europe (13 countries), high-income North America (2 countries) and high-income Oceania (2 countries)

For males in high-income Europe, North America and Oceania, we found a steep increase in the proportion of smoking-attributable mortality from the cohorts born 1870 up to about 1900–1910, when smoking-attributable mortality was the highest (Fig. 2). For high-income North America and Oceania, after reaching a peak for the 1910–1930 cohorts, there was a large drop in the proportion of smoking-attributable mortality in more recent cohorts. For Europe, the upsurge was followed by a stagnation period up to the most recent cohorts, where a steep drop was seen.

For females in high-income North America, the upsurge in smoking-attributable mortality was delayed by about 30 years relative to males (Fig. 2). At the highest ages, which are necessarily older cohorts, the peak in the proportion of smoking-attributable mortality does not seem to have been reached as yet, although some indication exists. For the younger ages, the peak was reached for the 1930 cohorts, with a steep decline after, interrupted only by the 1950 cohorts. For European females, smoking-attributable mortality increased less steeply and peaked later and lower than for North-American females. Any decline in smoking-attributable mortality in European females is seen in the most recent cohorts only, necessarily at younger ages. At higher ages, the proportion of smoking-attributable mortality is still on the increase. For Oceania, the pattern was similar to that of the Europe, but without any significant drop to date.

In terms of absolute levels of smoking-attributable mortality (rather than trends), smoking-attributable mortality was higher in males than for females in Europe for all age groups and all cohorts, even though for recent cohorts the absolute differences were small (Fig. 2). For the US and Oceania, smoking-attributable mortality was higher for males than for females for most cohorts and age groups, in particular those that drove recent changes. However, for recent cohorts at relatively young ages, smoking-attributable mortality was similar between sexes, even slightly higher for females than for males.

We produced an alternative version of Fig. 2 with age on the horizontal axis and each birth cohort represented by a line (Additional file 1: Figure S3), providing a complementary perspective of the smoking epidemic in the high-income regions.

**Fig. 3:**
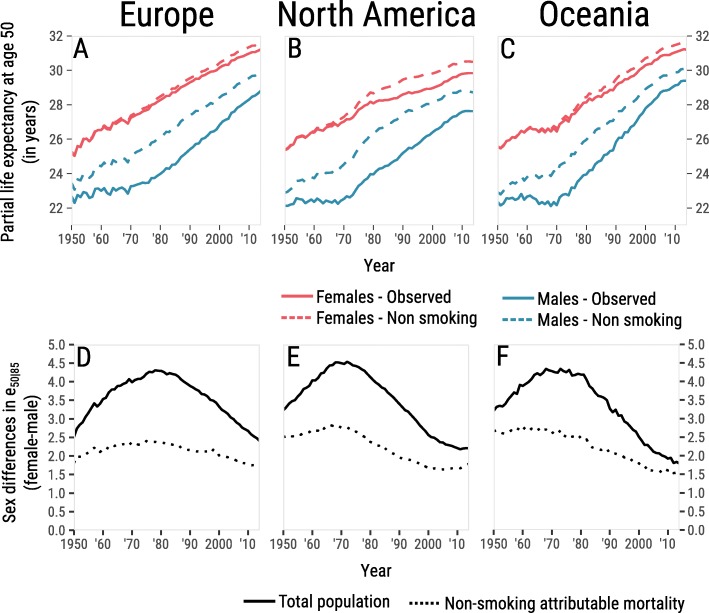
Upper panel: Historical development of period life expectancy between ages 50 and 85 (*e*_50 ∣ 85_, in years) for males (blue) and females (red) with the observed mortality rates (solid), and when smoking-attributable mortality was omitted (dashed), for the three studied regions. Lower panel: Sex differences in period *e*_50 ∣ 85_ with observed mortality (total population), and when smoking-attributable mortality was removed, for the three studied regions

The effect of removing smoking-attributable mortality on *e*_50 ∣ 85_ was similar for the three regions. For males, it increased gradually for the years ~ 1950–1970, to up to ~ 2 years of partial life expectancy (Fig. 3, top panels). This was particularly pronounced for Belgium, The Netherlands and the UK, while the increase was smallest for Sweden (Additional file 1: Figure S4). In the following years the effect of removing smoking-attributable mortality on *e*_50 ∣ 85_ remained more or less constant for some decades until approximately 1990. Afterward it declined to ~ 1 year for recent years. For females, the effect of removing smoking-attributable mortality on *e*_50 ∣ 85_ was negligible for the years 1950–1970. Afterwards it grew slowly but steadily to ~ 0.5 year. This was particularly pronounced in the US, but less so in Europe and Oceania.

Globally, over all three regions, the sex gap in *e*_50 ∣ 85_ was approximately 3 years in 1950, then increased to some 4.5 years around 1975, and afterwards decreased towards 2 years (Fig. 3, bottom panels). Omitting smoking-attributable deaths, the male-female difference in *e*_50 ∣ 85_ would have been approximately 2 years lower at its peak and much more constant over time than with smoking included (Fig. 3, bottom panels).

The contribution of smoking to male-female differences in life expectancy is now on the decline (Figs. 2 and 3). In some countries (Italy, New Zealand, Finland, Spain), this is due to declining smoking attributable mortality in males. In other countries (Sweden, Norway, Iceland), this is mainly due to increasing smoking-attributable mortality in females. Finally, there are countries (Canada, Austria, UK, US, Netherlands, Australia) where the decline is caused by an approximately equal contribution to each side of the *e*_50 ∣ 85_ gap (Additional file 1: Figure S5).

## Discussion

Our study advanced three main results. First, in all three high-income regions, smoking explained up to 50% of sex differences in period life expectancy between age 50 and 85 over the study period 1950–2015. Second, the decline in these sex differences since approximately 1980 is largely driven by smoking-attributable mortality. Third, whereas smoking-attributable mortality is still increasing for many older female cohorts, it is declining for females in the more recent cohorts in the US and Europe, as well as for males in all three regions.

The massive impact of smoking on mortality is in line with previous studies addressing smoking effects on mortality at the population level; it has been found for the United States [14], in European countries [12, 15, 30–35], and worldwide [36]. Smoking affects various causes of death, such as various forms of cancer, cardiovascular disease and multifarious diseases of the respiratory tract [37, 38]. Smoking also explains important differences in life expectancy between countries [39]. Finally, the historical trajectories of divergence between life expectancy with and without smoking-attributable mortality that we found are broadly similar to those previously found for specific countries [40].

### Insights into the smoking epidemic across cohorts

Our cohort-by-age analysis of high-income regions confirmed the mortality element of the smoking epidemic model [4, 5]: the increase in smoking-attributable mortality started later among females than for males, and resulted in a later peak at a lower level. Without constructing cohort profiles, we would not have been able to trace two additional important regularities of the smoking epidemic. First, while smoking-attributable mortality in older cohorts still increased, a precipitous decline in smoking-attributable mortality took place in recent cohorts at younger ages. Second, smoking-attributable mortality for males versus females converged across cohorts.

The continuous increase in smoking-attributable mortality in older female cohorts remains cause for concern. It is an essential feature of the smoking epidemic, though, that these deaths result from the high smoking prevalence of women decades ago. More encouraging is the decline in smoking-attributable mortality in recent cohorts at younger ages. Also, smoking prevalence has generally come down over the last decades in the studied regions. For example, in Australia smoking prevalence in females aged 15+ came down from 22.0% in 2000 to 12.4% in 2015 (for males, these numbers were 27 and 14.3%, respectively) [41].

In the same vein, current sex-specific smoking prevalence gives some indication of future sex-specific smoking attributable mortality. In 2015 smoking prevalence generally remained higher in males than in females. For example, in 2015 32% of French men smoked, versus 22% of females; in Germany, 25% of males smoked versus 17% of females; while in the US 14% of males smoked versus 12% of females [7]. Consequently, male smoking-attributable mortality is likely to remain (or become again) higher than female smoking-attributable mortality.

### The smoking epidemic and the sex gap in life expectancy

In 1950 the sex gap in period *e*_50 ∣ 85_ was 2.0–3.5 years. It subsequently grew to 4.3–4.5 years at the maximum around 1970–1980, and then decreased to 1.8–2.5 years in 2015 for the three regions. The rise, stagnation and decline in the sex differences in survival has been described in detail elsewhere [13, 16–18]. Smoking behaviour has been found to explain international differences in the life expectancy sex gap [42–44]. We here show that across high-income regions, almost no increase in the sex gap would have occurred without smoking-attributable mortality. Smoking-attributable mortality caused almost all the increase and most of the decrease in the sex gap over the study period.

Although the major part of the decline in the sex gap hitherto is caused by the steep drop in smoking-attributable mortality in males, more and more so this is also due to the increases in smoking-attributable mortality in females overall. For all-causes mortality, in contrast, it has been found that a reduction in the male-female life expectancy gap is, for most countries, due to men dying at lower rates, rather than women at higher rates [18, 45].

We suggest that we may not have seen the end of the narrowing in *e*_50 ∣ 85_ sex differences in these regions yet. To date, male smoking-attributable mortality generally still exceeds that of females. Meanwhile, trends are downward for males generally, while for females they are upwards for older cohorts. This suggests scope for further narrowing sex differences in *e*_50 ∣ 85_ in these regions. However, the extent to which this may happen seems limited because male smoking prevalence generally remains higher nowadays than female smoking prevalence (see above). Of course, smoking-attributable mortality is not the only factor that affects the sex gap, and there is evidence that mortality from some causes other than smoking may currently be widening the gap [16]. Still, smoking-attributable mortality could overwhelm the effect of mortality from other causes on the sex gap. This could happen especially in countries with a high proportion of women taking up smoking some decades ago, where smoking-attributable mortality for men and women could potentially cross over (e.g. U.K., Denmark and the Netherlands) [8], as we have found for the most recent cohorts in the US and Oceania.

### Limitations

One clear limitation of our study is the indirect calculation of the smoking-attributable deaths. Such a limitation is unavoidable: comparisons between different methods to estimate smoking-attributable mortality did not reveal a best-practice method [46, 47], and even if good estimates of smoking prevalence are available to potentially directly estimate smoking-attributable mortality, other factors like smoking intensity are often harder to measure and to take into account in direct estimates.

Since the PGW method [23] extrapolates the lung cancer rates of non-smokers from a US study to other countries, there may be a bias in our estimates for those other countries. Also, the PGW is based on study participants that are more likely than the US overall population to be Caucasian and middle class, and to have achieved a relatively high level of education [47]. However, previous analyses have shown that the indirect estimation by PGW resulted in roughly similar outcomes compared to other indirect estimation techniques [30, 31, 48], so we are confident that our results are broadly reliable. Results obtained making a modification to the PGW method proposed by Rostron [49], discussed in [50], are included in the Additional file 1. Making this modification would not have affected our main conclusion.

As a final limitation, our estimates of smoking-attributable mortality were smoothed over ages, which may lead to minor distortions. We do not expect that to be the case here due to relatively regular source data.

## Conclusion

While a sharp decrease in recent cohorts is a strong positive development, smoking-attributable mortality for both sexes has converged across cohorts to a level that remains high. Males and females dying of smoking at the same rate is not an end to the smoking epidemic. In high-income regions, smoking remains a major killer.

## Supplementary information

**Additional file 1.** Appendix.

## Data Availability

All data are publicly available from the Human Mortality Database (www.mortality.org) and the WHO Mortality Database (http://www.who.int/healthinfo/mortality_data/en/).

## Abbreviations

HMD: Human Mortality Database
ICD: International Statistical Classification of Diseases, Injuries and Causes of Death
PGW: Preston, Glei, Wilmoth
WHO: World Health Organization
