# Supplementary material for: Progression of the smoking epidemic in high-income regions and its effects on male-female survival differences: a cohort-by-age analysis of 17 countries
Source: BMC Public Health. 2020 Jan 10;20:39. doi: 10.1186/s12889-020-8148-4 (PMC6954612; doi:10.1186/s12889-020-8148-4)
Supplement: Supplementary file 1 — Additional file 1. Appendix. [file 12889_2020_8148_MOESM1_ESM.docx]

**Supplemental Material**

## Missing data

We report (our handling of) missing data according to the guidelines set out by Sterne J  et al. Multiple Imputation for Missing Data in Epidemiological and Clinical Research: Potential and Pitfalls. BMJ, 338, b2393 2009 Jun 29. PMID: 19564179.

Missing item: *cause-specific death rates, 3 years for each sex, Portugal*. The reason for missingness was that Portugal used a special classification system during that period, which cannot be reliably translated to ICD-10. Given this known reason, missingness was unlikely to be related to the number of lung cancer deaths in these years. Overall death rates (which are known) did not deviate from the trend in the years in which cause-of-death information was missing. Variables used in imputation where the cause-specific death rates for Portugal in the available years. Thus, we assumed that the time trend in the years 2004-2006 was a smooth continuation of the time trend before and after 2004-2006, and so that the cause-specific death rates for those years were missing completely at random. Population size of Portugal is small compared with the rest of high-income Europe and we imputed for three years only, so that this imputation in no way affects our overall conclusions (which take a large perspective). The missForest algorithm took 10 iterations until convergence.

## Model fit

To assess model fit, we performed an analysis of deviance and visually inspected the residuals. For females, the PGW model had a null deviance of 1,768,593 on 11,103 degrees of freedom versus a residual deviance of 11,095 on 10,980 degrees of freedom. This gives a p-value for the model of p<10^-20^ and a Chi-squared goodness-of-fit test p-value of 0.22. All parameters were significant (p<10^-7^). For males, the PGW model had a null deviance of 1,587,178 on 11,103 degrees of freedom versus a residual deviance of 11,218 on 10,980 degrees of freedom. This gives a p-value for the model of p<10^-20^ and a Chi-squared goodness-of-fit test p-value of 0.055. All parameters were significant (p<10^-7^). The residuals were symmetric (Figures S1 and S2).

**
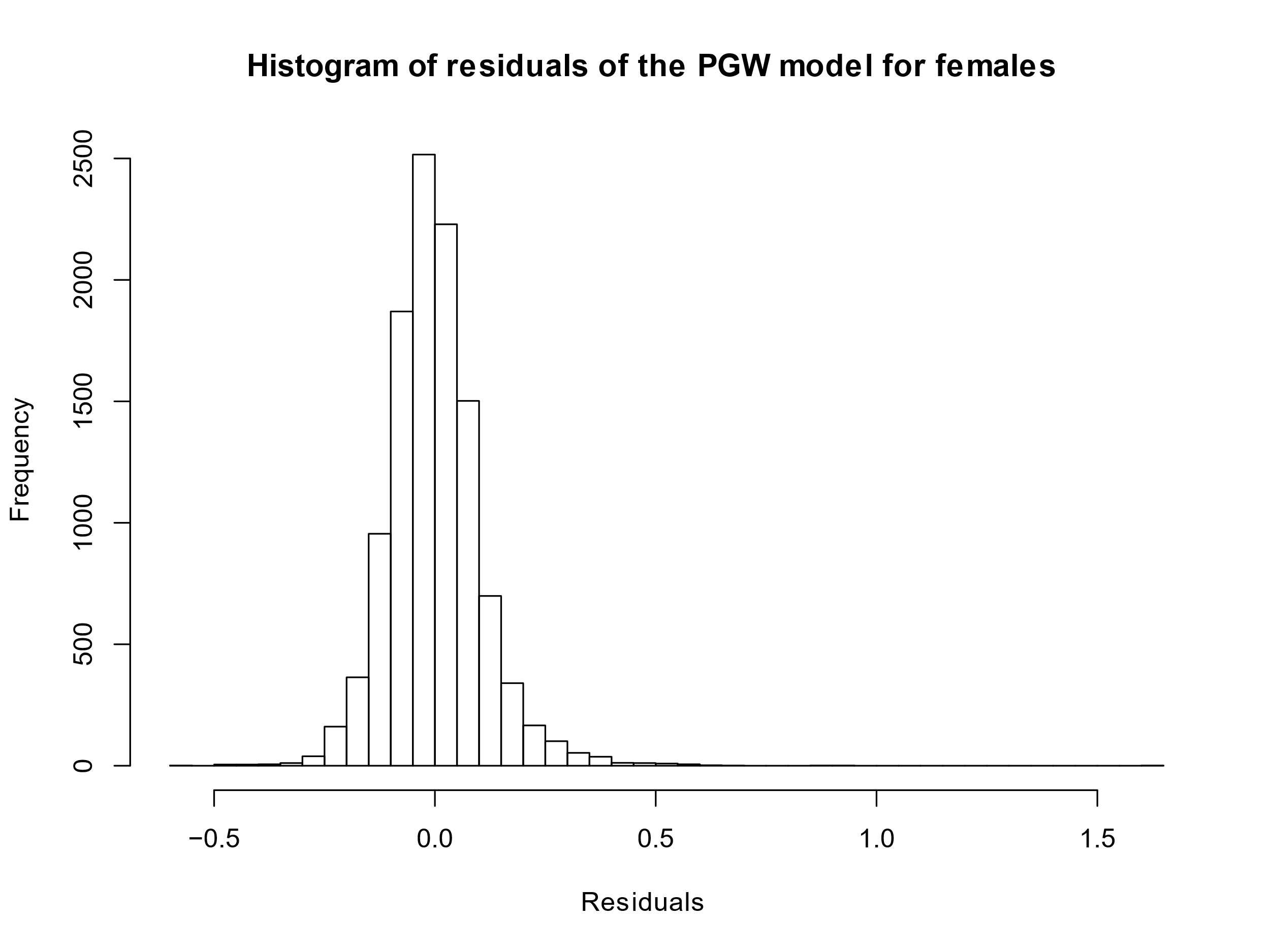
**

Figure S1. Histogram of residuals of the Preston-Glei-Wilmoth model. Females.

**
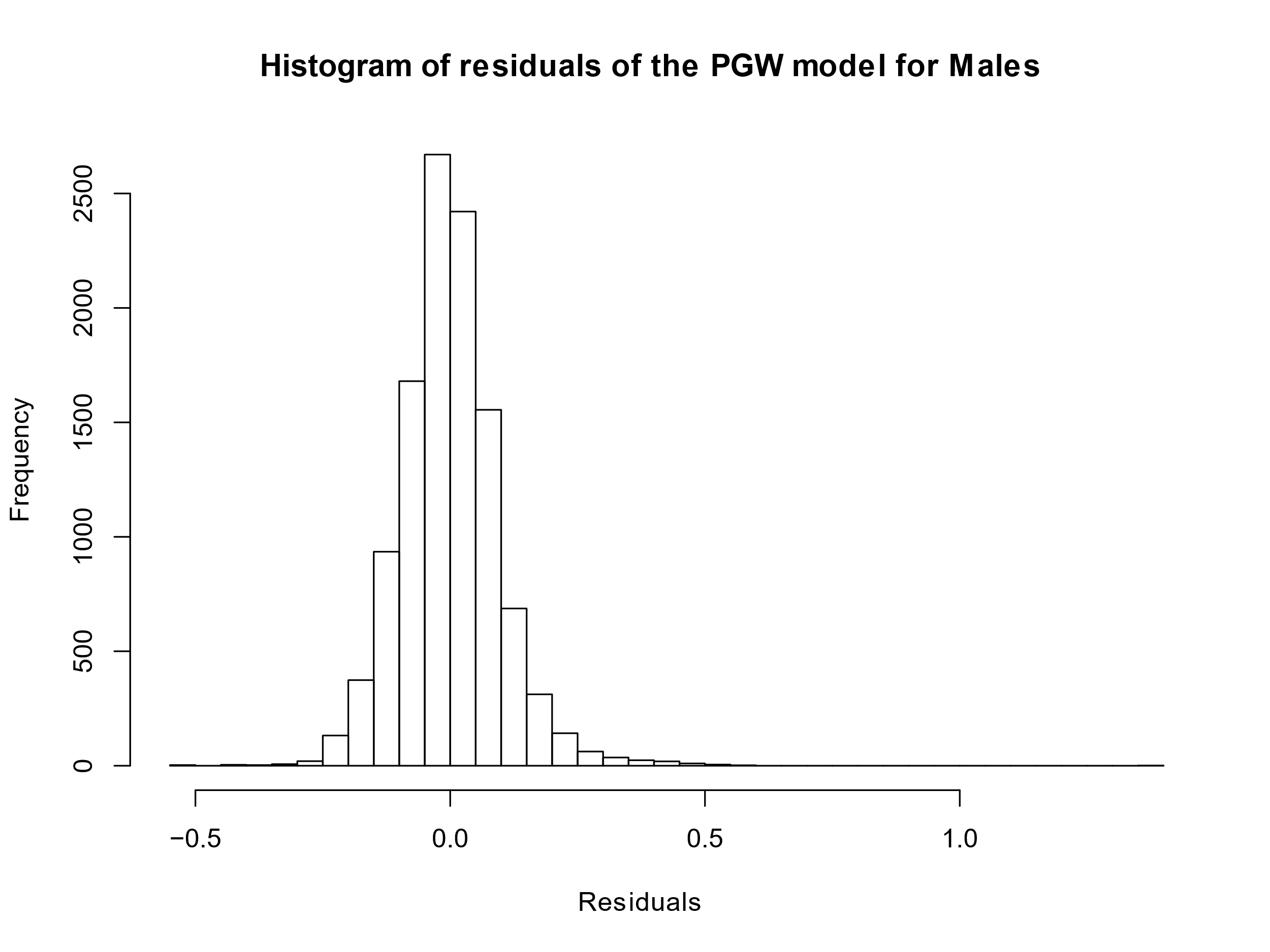
**

Figure S2. Histogram of residuals of the Preston-Glei-Wilmoth model. Males.

## Alternative Figure 2


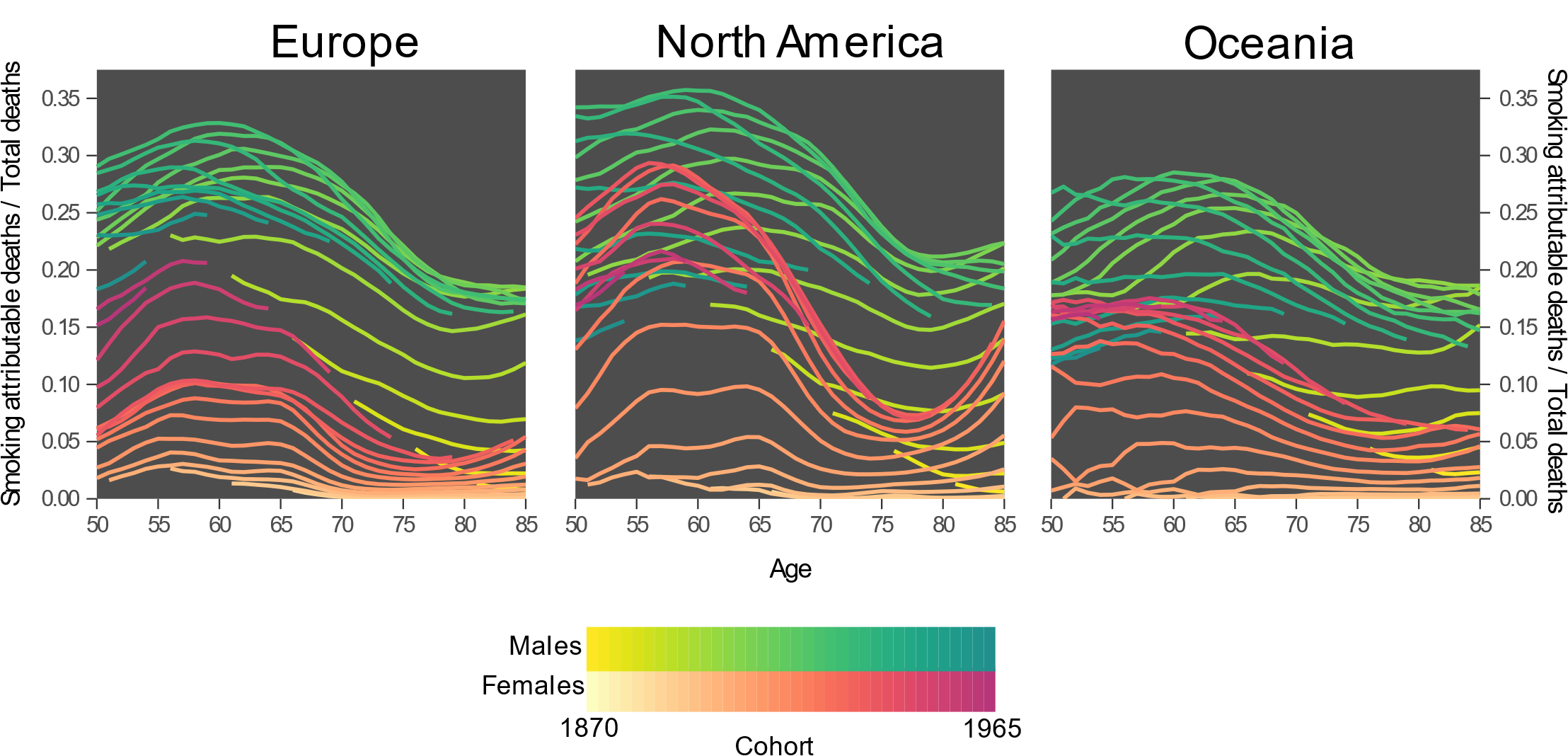


Figure S3. Proportion of overall mortality that is attributed to smoking. This is an alternative representation of the results shown in Figure 2. Each birth cohort is on a single vertical line. For males, cohorts are shaded from yellow (earlier cohorts) to turquoise (younger cohorts). For females, cohorts are shaded from beige (earlier cohorts) to fuchsia (younger cohorts). Results given for high-income Europe (13 countries), high-income North America (2 countries) and high-income Oceania (2 countries).

## Per-country trends


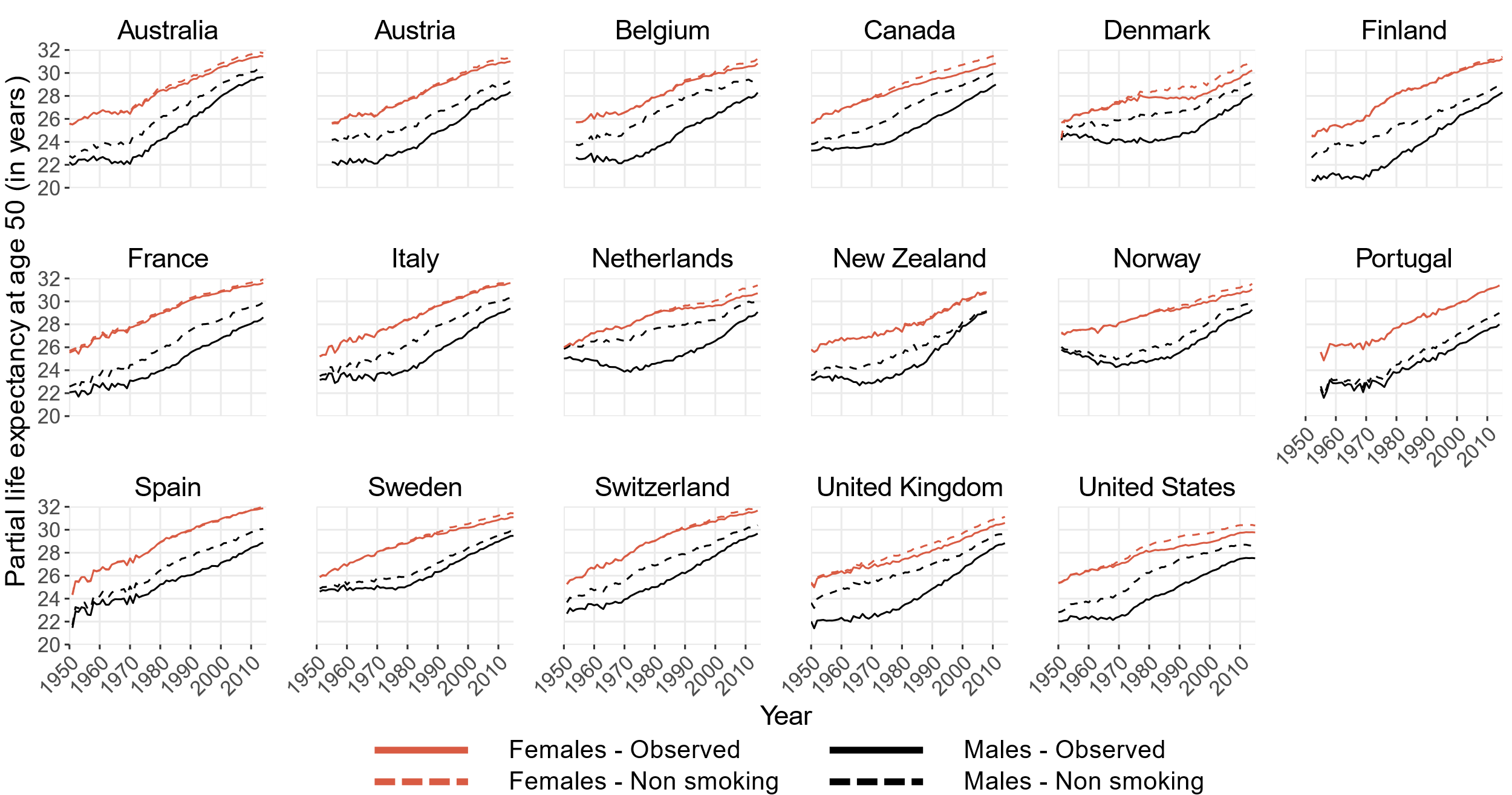


Figure S4. Historical development of period partial life expectancy between ages 50 and 85 ($e_{50|85}$, in years) for males (in black) and females (in red) with the observed mortality rates (solid), and when smoking-attributable mortality was omitted (dashed). Results shown for individual countries


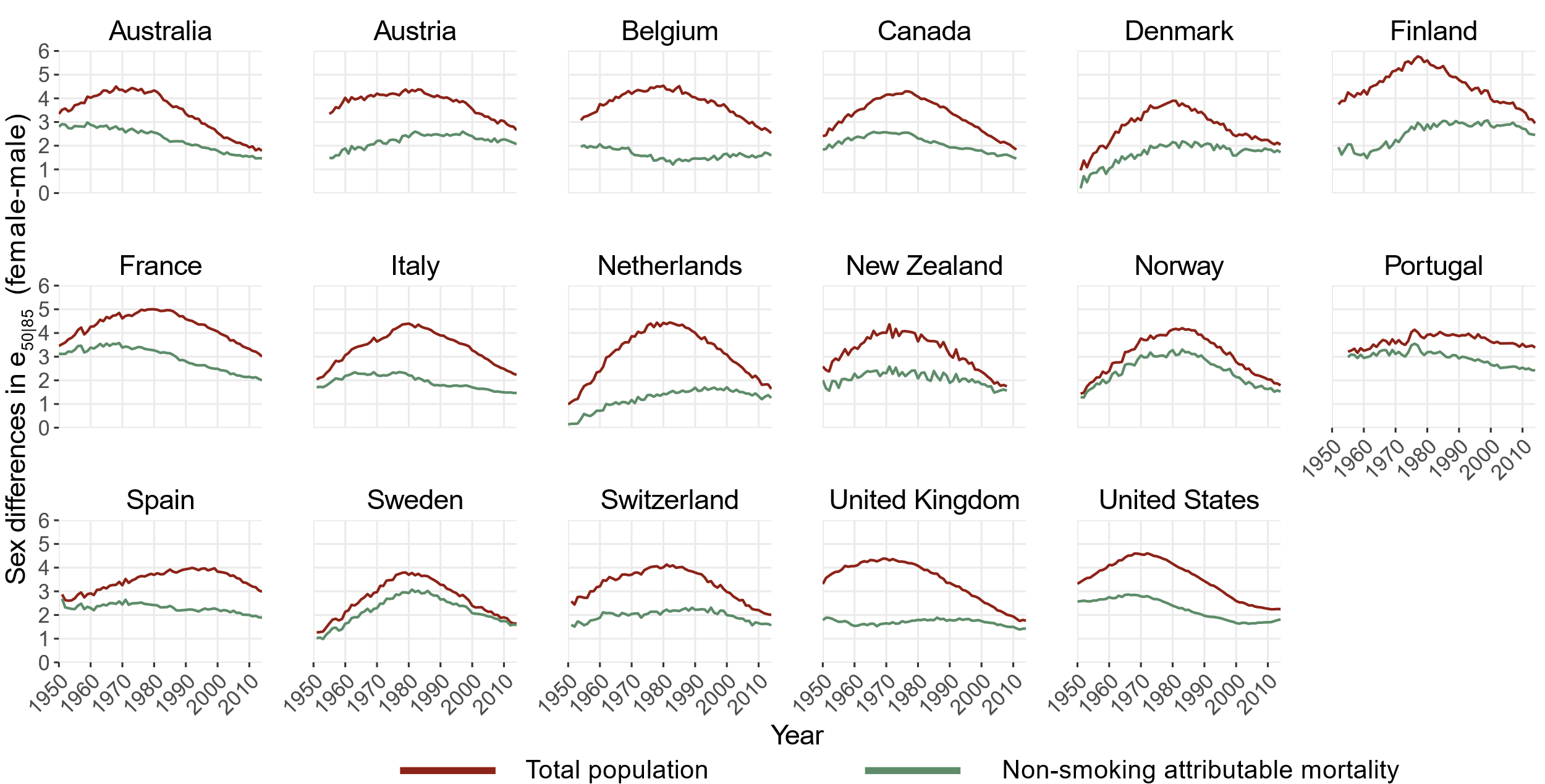


Figure S5. Sex differences in period $e_{50|85}$ with observed mortality (total population), and when smoking-attributable mortality was removed. Results shown for individual countries.

## Age-period interaction

The PGW method has been criticized for producing results that are different from earlier results produced by the Peto-Lopez method [49 of main text], especially for older females. The modification of the PGW method by Rostron produced lower smoking-attributable mortality for ages 80 and over, which were more in line with earlier results [49 of main text]. On the other hand, the reason for introducing the PGW method was that the authors felt that the Peto-Lopez method unduly relied on relative risk estimates [23, 50 of main text]. Differing from earlier results is therefore not necessarily a weakness of the method. Both methods produce estimates, not truth, but our estimates certainly seem reasonable. Because the same method has been used over time, the time trends are most likely less affected than the levels themselves. In our analysis, the modification proposed by Rostron led to lower results for higher ages for both sexes (Figure S6 below). This would not have changed the main conclusions of our study.


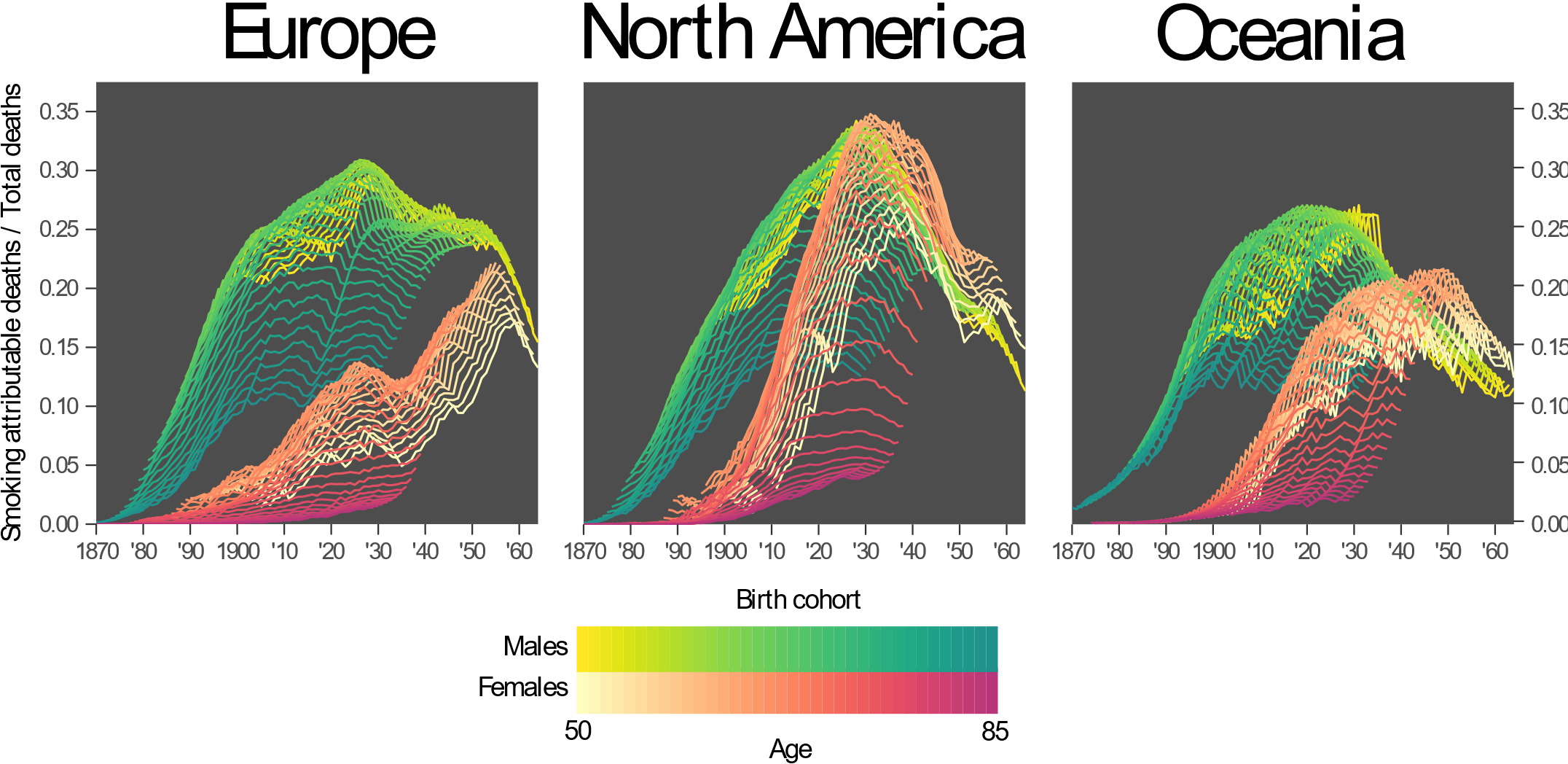


Figure S6. Similar to Figure 2 in the main text, but with a modification proposed by Rostron (ref 49 of the main text). An interaction term is added between age groups and time to the regression equation used by PGW (ref 23 of the main text).
